# Management of a small bowel hemangioma causing intussusception in an infant: A rare case report and literature review

**DOI:** 10.1016/j.ijscr.2024.110108

**Published:** 2024-07-31

**Authors:** Imen Helal, Anis Hasnaoui, Aida Daïb, Raja Jouini, Fatma Khanchel, Ashraf Chadli Debbiche

**Affiliations:** a Faculty of Medicine of Tunis, Tunis El Manar University, Rue Djebal Lakhdar, 1006 Tunis, Tunisia; b Department of Pathology, Habib Thameur Hospital, Tunisia; c Department of Pediatric Surgery, Habib Thameur Hospital, Tunisia

**Keywords:** Hemangioma, Small bowel, Intestinal intussusception, Children, Case report

## Abstract

**Introduction:**

Hemangiomas of the small intestine are rare and usually present in young people. They are very difficult to diagnose preoperatively. We report a rare case of mixed intestinal hemangioma (IH) causing intussusception in a pediatric patient.

**Case presentation:**

A 3-month-old girl, with no prior medical or surgical history, was admitted with rectal bleeding and paroxysmal crying due to intermittent abdominal pain. An urgent abdominal ultrasound revealed ileo-ileal intussusception. Operative findings confirmed the intussusception, and a segmental resection of the intussuscepted jejunum was performed. Histopathological examination found a mixed hemangioma. The postoperative course was uneventful.

**Discussion:**

Clinical presentation may include intestinal bleeding leading to anemia, obstruction, intussusception and perforation. Intussusception caused by small bowel hemangioma is extremely rare. Notably, we didn't find any cases of small bowel hemangioma revealed by intussusception in children. The main treatment for hemangiomas is surgical resection of the affected segment. No evidence in the literature on postoperative recurrence of hemangiomas.

**Conclusion:**

Intussusception secondary to intestinal hemangiomas is extremely rare. Preoperative diagnosis is challenging as they are often undetectable with traditional techniques. Enhanced awareness and understanding of this condition can facilitate earlier diagnosis and improve management outcomes.

## Highlights

• Jejunal intussusception secondary to an intestinal hemangioma is extremely rare.
• No similar cases were reported in children.
• Surgery is the safest option for managing intestinal intussusception when there is suspicion of perforation or necrosis.
• Hemangiomas are difficult to diagnose preoperatively with traditional techniques like upper and lower endoscopies.
• The main treatment for hemangiomas is surgical resection of the affected segment.

## 1 Introduction

Intestinal hemangiomas (IH) are rare, accounting for only 0.05 % of all intestinal neoplasms [1]. These growths typically occur in young people, with no preference for either sex [2]. The jejunum is the most affected site in the gastrointestinal tract [2]. Common symptoms include abdominal pain, bleeding, and chronic anemia [3]. Intussusception caused by a small bowel hemangioma is extremely rare. Here, we report a case of a mixed hemangioma of the jejunum in a 3-month-old girl admitted for intussusception. This case report has been reported in line with the SCARE Criteria [4].

## 2 Case presentation

**Fig. 1:**
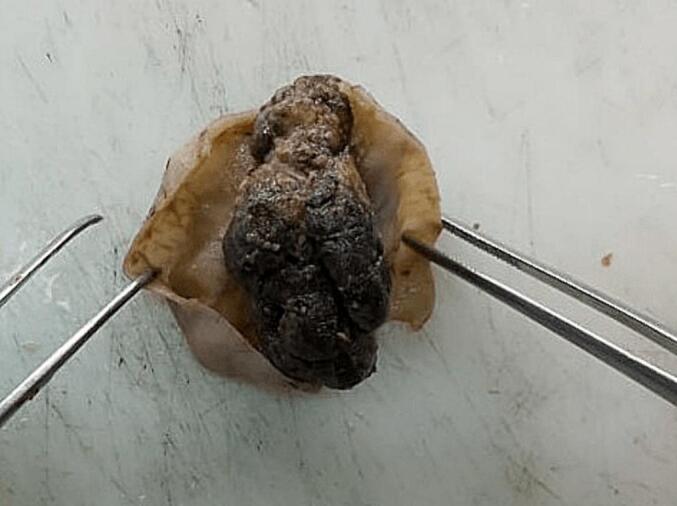
Macroscopic examination: A large polypoid pedunculated lesion measuring 4.6 × 2.1 × 1.5 cm in the lumen.

**Fig. 2:**
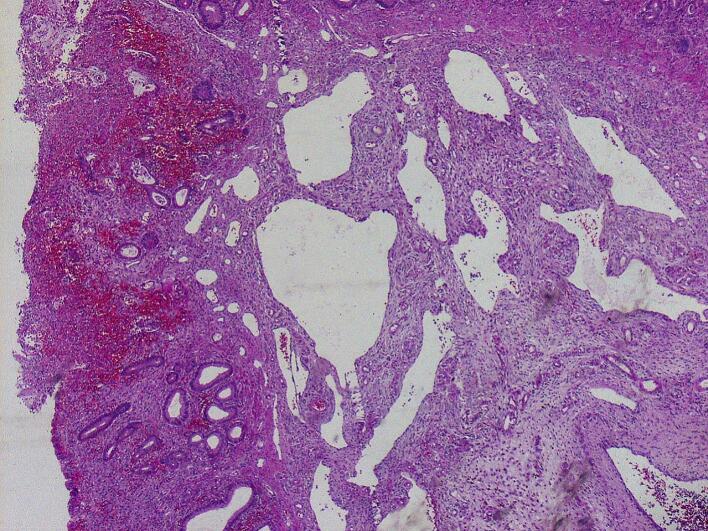
Microscopic examination: multiple various blood-filled spaces which were lined by thin endothelial cells and separated by scant inflammatory and connective tissue stroma.

A 3-month-old girl, with no prior medical or surgical history, was admitted with rectal bleeding and paroxysmal crying due to intermittent abdominal pain. Clinical examination revealed a mild fever of 38 °C, tachycardia with a heart rate of 180 beats per minute, and signs of dehydration including skin turgor, sunken eyes and depressed fontanelle. The patient also exhibited abdominal bloating, tenderness and hematochezia. A non-contrast abdominal X-ray demonstrated air-fluid levels in the small bowel. An urgent abdominal ultrasound revealed a jejunal intussusception. The patient's general condition rapidly worsened, leading to fatigue and pallor. Given the high probability of necrosis associated with the jejunal intussusception we opted for surgery rather than radiographic reduction, which is not commonly performed for proximal intussusception in our department. After a short period of resuscitation, the patient underwent a midline laparotomy with a presumptive diagnosis of intestinal intussusception. Operative findings confirmed the intussusception, and a segmental resection of the intussuscepted jejunum was performed, followed by an end-to-end anastomosis. Macroscopic examination revealed a large polypoid pedunculated lesion measuring 4.6 × 2.1 × 1.5 cm in the lumen (Fig. 1). Hematoxylin and eosin staining of representative sections showed multiple blood-filled spaces lined by thin endothelial cells and separated by scant inflammatory and connective tissue stroma. The overlying mucosa displayed hyperplastic glands (Fig. 2). Immunohistochemical analysis showed positivity for CD31. The final diagnosis was a mixed hemangioma causing intussusception of the jejunum. The postoperative course was uneventful, and the infant was discharged 7 days after surgery.

## 3 Discussion

Hemangioma is a benign vascular proliferation and should be distinguished from vascular malformation which is a lesion with structural anomalies and not a true tumor. IH are rare and account for 7–10 % of all benign neoplasms of small bowel [1]. IH may be solitary or multiple as a manifestation of Maffucci syndrome, Klippel-Trenaunay syndrome, disseminated neonatal hemangiomatosis, or blue rubber bleb nevus syndrome [5]. A search of PubMed database was performed to find studies on solitary hemangioma of small bowel in patients under 16 years, published before December 2023. We identified 23 cases in total [1,3,[6], [7], [8], [9], [10], [11], [12], [13], [14], [15], [16], [17], [18], [19], [20], [21], [22], [23], [24], [25], [26]]. Table I summarizes the patients' data. Clinically, gastrointestinal hemangiomas are symptomatic in 90 % of cases [27]. Clinical presentation includes abdominal pain, bleeding, chronic symptoms of anemia, obstruction, perforation, intramural hematoma, and intussusception [8]. Intussusception secondary to a small bowel hemangioma is extremely rare. Notably, we didn't find any cases of small bowel hemangioma revealed by intussusception in children, in the English literature. Very few cases causing intussusception were described in adults [28,29].

**Table I:** Small bowel hemangiomas in infants reported in the literature.

Ref (author, year)	Age	Sex	Presentation	Size (cm)	Type	Location	Treatment
Copple et Kingsbury, 1961 [6]	5 years	M	Hematemesis anemia	1,5 cm (2 lesions)	Cavernous	Jejunum	Surgical resection
McGaughey et Hosie, 1966 [7]	6 days	M	Vomiting	Not given	Cavernous	50 cm from the ileocaecal junction	Surgical resection
Hyun et al., 1969 [8]	12 years	M	Anemia melena	Not given	Cavernous	Not given	Surgical resection
Elefant et al., 1970 [9]	1 day (newborn)	M	Bilious vomiting, intestinal obstruction	Not given	Arterio-capillary hemangioma	Ileum	Surgical resection
Fawcett et al., 1986 [10]	3 years	F	Anemia	Not given	Cavernous	Jejunum	Surgical resection
Boyle et al., 1993 [1]	14 years	M	Anemia		Cavernous	Jejunum	
Sakaguchi et al., 1998 [11]	11 years	M	Anemia Abdominal pain	6 cm	Cavernous	Ileum	Surgical resection
Magnano et al., 2005 [12]	13 years	M	Fatigue, weakness, anemia	2 cm	Cavernous	Ileum	Surgical resection
Kavin et al., 2006 [13]	2.5 years	F	Melenic stools	Not given	Mixed capillary hemangioma	Jejunum	Surgical resection
Dudesek et al., 2006 [14]	Young	F	Haemoperitoneum	Not given	Cavernous	Not given	
Jones et al., 2007 [15]	7 years	F	Lethargy, anorexia, abdominal pain	2 cm	Cavernous	Jejunum	Surgical resection
Pinho et al., 2008 [16]	9 years	F	Fatigue, dizziness, anemia, melena	2.5 cm	Cavernous	Ileum	Surgical resection
Abdul Aziz DA, 2011 [3]	6 years	F	Intestinal obstruction after abdominal trauma	Not given (huge mass)	Cavernous	Ileum	Surgical resection
Pera et al., 2012 [17]	16 years	M	Anemia, palpitation, fatigue	4.3 cm	Cavernous	Jejunum	Surgical resection
Park, 2012 [18]	2 years	M	Intestinal bleeding	10 cm	Cavernous	Jejunum	Surgical resection
Turcotte et al., 2012 [19]	16 years	F	Anemia	2.5 cm	Capillary	Jejunum	Surgical resection
Shukri et al., 2013 [20]	27 days	M	Acute intestinal obstruction	Not given	Capillary	Ileum	Surgical resection
Papprella et al., 2014 [21]	Not given	Not given	Not given	Not given	Not given	Jejunum	Surgical resection
Han et al., 2014 [22]	13 months	M	Melena, non-bilious vomiting	5 cm	Cavernous	Jejunum	Surgical resection
Bae et al., 2015 [23]	13 years	M	anemia, nausea, dizziness	5.2 cm	Cavernous	Jejunum	Surgical resection
Coleman et al., 2018 [24]	2 years	F	Anemia	4.5 cm	Not given	Not given	Surgical resection
Khan et al., 2019 [25]	2 years	M	Bilious emesis, anorexia	9.6 cm	Cavernous	Jejunum	Surgical resection
Fu et al., 2020 [26]	5 years	F	Abdominal pain, nausea, vomiting, anemia	10 cm	Not given	Ileum	Surgical resection

Hemangiomas are difficult to diagnose preoperatively with traditional techniques like upper and lower endoscopies, but advanced imaging modalities such as capsule endoscopy, double balloon enteroscopy (DBE), computed tomography and magnetic resonance imaging are available options for investigating small bowel lesions. In an emergency setting like ours, the final diagnosis is typically made through histologic examination. Macroscopically, IH is a bluish-purple lesion. Histologically, the lesion is composed of blood-filled spaces of variable sizes and shapes. The lumens are filled with blood cells, lined by thin endothelial cells, and separated by connective tissue stroma. According to the size of affected vessels, hemangiomas are histologically classified as cavernous, capillary, or mixed type [1]. The main treatment for hemangiomas is surgical resection of the affected segment. Gastrointestinal hemangiomas usually have a satisfying prognosis, and there is no evidence in the literature on the recurrence of hemangiomas [26].

## 4 Conclusion

Intestinal hemangiomas are rare, and those presenting as intussusception are even rarer. Preoperative diagnosis is challenging as they are often undetectable with traditional techniques. Enhanced awareness and understanding of this condition can facilitate earlier diagnosis and improve management outcomes.

## Abbreviations

IH: intestinal hemangioma

## Consent for publication

Written informed consent was obtained from the parents for publication and any accompanying images after disidentification. A copy of the written consent is available for review by the Editor-in-Chief of this journal on request.

## Ethical approval

Ethical approval was deemed unnecessary by the institutional ethics committee of Habib Thameur Hospital, Tunis, Tunisia, as the paper is reporting a single case that emerged during normal practice. Ethical approval is not required in our institution for case reports.

## Funding

This research did not receive any specific grant from funding agencies in the public, commercial, or not-for-profit sectors.

## Author contribution

Imen Helal: Conceptualization, writing-Original draft preparation. Anis Hasnaoui: Writing-Reviewing and Editing. Aida Daïb: Data curation. Raja Jouini: Data curation. Fatma Khanchel: Data interpretation. Ashraf Chadli Debbiche: Reviewing and Editing. All authors read and approved the final manuscript.

## Guarantor

Anis Hasnaoui.

## Conflict of interest statement

The authors declare that they have no competing interests.
